# Clinical significance of the largest histopathological metastatic lymph node size for postoperative course of patients undergoing surgery for gastric cancer

**DOI:** 10.3389/fsurg.2023.1105189

**Published:** 2023-02-17

**Authors:** Sinan Omeroglu, Selcuk Gulmez, Pinar Yazici, Uygar Demir, Onur Guven, Emir Capkinoglu, Orhan Uzun, Aziz Serkan Senger, Erdal Polat, Mustafa Duman

**Affiliations:** ^1^ Department of General Surgery, University of Health Sciences Sisli Hamidiye Etfal Research and Training Hospital, Istanbul, Türkiye; ^2^ Department of Gastrointestinal Surgery, University of Health Sciences Kosuyolu High Specialization Education and Research Hospital, Istanbul, Türkiye

**Keywords:** gastric cancer, lymph node metastasis, lymph node size, survival, postoperative complication

## Abstract

**Aim:**

The aim of this study was to investigate the effect of the largest metastatic lymph node (MLN) size on postoperative outcomes of patients with stage II-III gastric cancer (GC).

**Methods:**

A total of 163 patients with stage II/III GC who underwent curative surgery were included in this single-center retrospective study. The lymph nodes were counted, each lymph node was analyzed for metastatic involvement by histopathological examination, and the diameter of the largest metastatic lymph node was recorded. The severity of postoperative complications was assessed by Clavien–Dindo classification system. Two groups of 163 patients were defined according to ROC analysis with cut-off value of histopathologically maximum MLN diameter. A comparative analysis of demographic and clinicopathological characteristics of the patients and their postoperative outcomes were performed.

**Results:**

The median hospital stay was significantly longer in patients with major complications compared to patients without major complications [18 days (IQR: 13–24) vs. 8 days (IQR: 7–11); (*p* < 0.001)]. The median MLN size was significantly larger in deceased patients compared to survived [1.3 cm (IQR: 0.8–1.6) vs. 0.9 cm (IQR: 0.6–1.2), respectively; (*p* < 0.001)]. The cut-off value of MLN size predicting mortality was found as 1.05 cm. MLN size ≥1.05 cm had nearly 3.5 times more negative impact on survival.

**Conclusions:**

The largest metastatic lymph node size had a significant association with survival outcomes. Particularly, MLN size over 1.05 cm was associated with worse survival outcomes. However, the largest MLN was not shown to have any effect on major complications. Further prospective and large-scale studies are required to draw more precise conclusions.

## Introduction

Gastric cancer (GC) is the third most common cause of cancer-related deaths worldwide. Surgery is the gold standard for curative treatment of GC (1). Following surgical resection, examination of lymph nodes (LNs) are important for accurate staging, postoperative treatment approach, clinical follow-up and prognosis. LN metastasis plays a key role in the recurrence and long-term survival of the gastric cancer patients undergoing surgery (2, 3). D2 LN dissection and the number of metastatic LNs are well-known prognostic factors. In addition, the number of harvested LN and MLN ratio are important prognostic factors (3, 4). Eighth Edition of The American Joint Committee on Cancer (AJCC) Staging Manual is currently used for pathological examination (5). In this TNM classification, N staging is done by the number of metastatic lymph nodes (MLNs), neither MLN size nor MLN ratio is considered. Similar to the LN rate, the effect of the size of the positive LN on the pathological stage, clinical follow-up, postoperative treatment approach, and prognosis are not taken into account in this staging system.

Chen et al. reported that tumor size can be included in AJCC staging, considering that it may have different prognostic roles in gastric cancer at different stages (6). In some series, it has been shown that MLN size is effective in the determination of the prognosis and it provides valuable support to the classification systems in patients with gastrointestinal malignancies, including colon and esophageal cancer (7, 8). However, there are limited number of reports investigating the relationship between the largest MLN size, prognosis and survival in gastric cancer (9, 10). The role of MLN size in the postoperative period of the gastric cancer patients remained a serious gap in the literature. Furthermore, to our knowledge, there is no research in the literature evaluating the relationship between metastatic largest LN size and postoperative complications in patients with GC. We aimed to investigate the effect of histopathologically determined metastatic largest LN size on postoperative outcomes in patients with Stage II-III GC.

## Materials and methods

This single-center, retrospective study was conducted at the Department of Gastroenterological Surgery, University of Health Sciences Kosuyolu High Specialization Education and Research Hospital, Istanbul, Turkey. The study was carried out in accordance with the Helsinki Declaration and local laws and regulations. This study was approved by the ethical committee of Kosuyolu High Specialization Education and Research Hospital with an IRB number: 2020/14/404.

**Figure 1:**
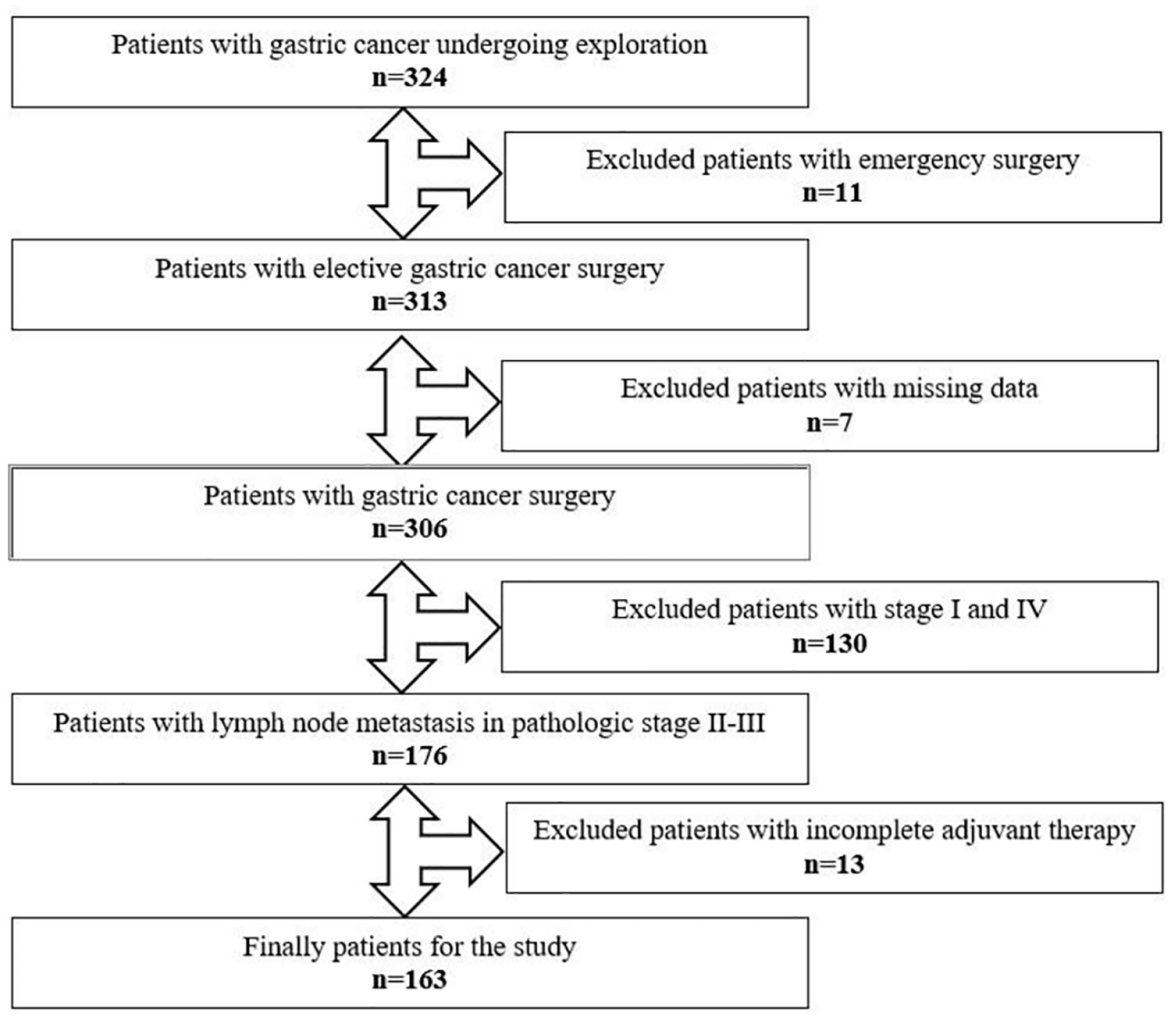
Flowchart of the inclusion.

Between December 2006 and December 2019, medical records of 324 patients who underwent gastric cancer surgery were retrospectively reviewed and data of 163 eligible patients were enrolled in the study (Figure 1). Patients aged over 18 who underwent a curative surgery for TNM stage II or III GC were considered eligible for this study. All patients underwent open total or subtotal gastrectomy with D2 lymphadenectomy. Patients who underwent emergency surgery, had immunodeficiency or lymphoproliferative disease and had taken immunomodulatory drugs were excluded. Also, patients whose adjuvant chemotherapy was not completed were not included into the study.

Data regarding the patients' age, gender, comorbidity status, presence/absence of lymphovascular and perineural invasions (LVI and PNI), tumor histological grade, tumor size and location, total number of harvested LNs and metastatic LNs, size of the largest MLN, length of hospital stay, postoperative complications, overall survival (OS), neoadjuvant treatment status were recorded. The Clavien-Dindo classification was used to analyze postoperative complications, and grade III or higher complications were defined as major complications (11).

Adjuvant chemotherapy was given to all patients with a pathological stage II and III gastric cancer with LN metastases. DCF (Docetaxel, cisplatin, 5-fluorouracil) or FLOT (5-fluorouracil, leucovorin, oxaliplatin, docetaxel) regimens were given as both neoadjuvant and adjuvant chemotherapy.

The software IBM® SPSS® (Statistical Package for the Social Sciences) version 23 (IBM Corp. Armonk, NY, USA) was used for statistical analysis. Qualitative data were presented as frequency and percentage. The distribution of numerical data was performed using the Kolmogorov–Smirnov test with the non-normal distribution results. Quantitative data were given as median with Interquartile Range (IQR). The association of major complications and survival with categorical variables was analyzed using Chi-square, Fisher's exact tests, and Likelihood ratio. The Mann–Whitney-*U* test was used to examine whether major complications and survival were related to age, metastatic lymph node size, and length of hospital stay. The Kaplan–Meier method and the log-rank test were used to conduct the survival analyses of the metastatic lymph node size. Further, multivariate Cox regression analyses were performed to examine role of the metastatic lymph node size in predicting mortality. A *p*-value of less than 0.05 was defined as statistically significant.

## Results

Patients' demographic and clinicopathologic characteristics considering the major complications and survival status were presented in Table 1. The median hospital stay was 18 (IQR: 13–24) days in patients with major complications, while it was 8 (IQR: 7–11) days in patients without major complications (*p* < 0.001). The median age of deceased patients was significantly higher than those who survived (63 [IQR: 57–69] vs. 57 [IQR: 50–65], respectively, *p* = 0.005). Both pT stage and pN stage were significantly higher in the deceased patient group (*p* = 0.012 and *p* = 0.026, respectively). The median MLN size was significantly larger among the deceased patients compared with the survived [1.3 cm (IQR: 0.8–1.6) vs. 0.9 cm (IQR: 0.6–1.2); (*p* < 0.001)]. The frequency of lymphovascular invasion and perineural invasion was also significantly higher in deceased patients (*p* = 0.043 vs. *p* = 0.017, respectively).

**Table 1:** Patients’ demographic and clinicopathologic characteristics and the effect of variables on major complications and survival status.

Variables *n* (%)		Major Complication		*p-value*	Survival		*p-value*	5-year OS^e^
		No *n* = 139 (85.3%)	Yes *n* = 24 (14.7%)		Exitus *n* = 111 (68.1%)	Alive *n* = 52 (31.9%)		
Age, years, median (IQR)		62 (52–68)	64 (59–70)	0.148^a^	63 (57–69)	57 (50–65)	0.005^a^	–
Gender	Male	105 (86.1%)	17 (13.9%)	0.624^b^	80 (65.6%)	42 (34.4%)	0.233^b^	34.8%
	Female	34 (82.9%)	7 (17.1%)		31 (75.6%)	10 (24.4%)		28.5%
CCI	0–2	107 (86.3%)	17 (13.7%)	0.515^b^	87 (70.2%)	37 (29.8%)	0.314^b^	31.1%
	≥3	32 (82.1%)	7 (17.9%)		24 (61.5%)	15 (38.5%)		40.1%
BMI, kg/m^2^	<30	112 (84.8%)	20 (15.2%)	0.503^c^	93 (70.5%)	39 (29.5%)	0.183^b^	31.1%
	≥30	27 (87.1%)	4 (12.9%)		18 (58.1%)	13 (41.9%)		43.1%
Neoadjuvant	No	79 (82.3%)	17 (17.7%)	0.198^b^	58 (60.4%)	38 (39.6%)	0.012^b^	44.5%
	Yes	60 (89.6%)	7 (10.4%)		53 (79.1%)	14 (20.9%)		15.3%
Surgery	Total	71 (84.5%)	13 (15.5%)	0.780^b^	55 (65.5%)	29 (34.5%)	0.459^b^	36.8%
	Subtotal	68 (86.1%)	11 (13.9%)		56 (70.9%)	23 (29.1%)		29.9%
pT stage	T1/T2	13 (76.5%)	4 (23.5%)	0.225^c^	7 (41.2%)	10 (58.8%)	0.012^b^	58.8%
	T3/T4	126 (86.3%)	20 (13.7%)		104 (71.2%)	42 (28.8%)		30.2%
pN stage	N1	41 (80.4%)	10 (19.6%)	0.405^b^	33 (53.8%)	18 (46.2%)	0.026^b^	44.2%
	N2	33 (84.6%)	6 (15.4%)		21 (64.7%)	18 (35.3%)		37.7%
	N3	65 (89.0%)	8 (11.0%)		57 (78.1%)	16 (21.9%)		25.1%
MLN size, cm, median (IQR)		1.2 (0.7–1.5)	1.0 (0.6–1.4)	0.163^a^	1.3 (0.8–1.6)	0.9 (0.6–1.2)	<0.001^a^	–
LVI	No	27 (84.4%)	5 (15.6%)	0.531^c^	17 (53.1%)	15 (46.9%)	0.043^b^	44.5%
	Yes	112 (85.5%)	19 (14.5%)		94 (71.8%)	37 (28.2%)		30.4%
PNI	No	29 (82.9%)	6 (17.1%)	0.649^b^	18 (51.4%)	17 (48.6%)	0.017^b^	46.1%
	Yes	110 (85.9%)	18 (14.1%)		93 (72.7%)	35 (27.3%)		29.5%
Differentiation	Well	2 (50.0%)	2 (50.0%)	0.159^d^	3 (75.0%)	1 (25.0%)	0.673^d^	–
	Moderately	44 (89.8%)	5 (10.2%)		31 (63.3%)	18 (36.7%)		39.6%
	Poorly	93 (84.5%)	17 (15.5%)		77 (70.0%)	33 (30.0%)		31.2%
LOS, days, median (IQR)	8 (7–11)	18 (13–24)	<0.001^a^	9 (7–12)	8 (7–17)	0.717^a^	–

^a^ Mann–Whitney *U* test.

^b^ Pearson's Chi-Square test.

^c^ Fisher's exact test.

^d^ Likelihood ratio.

^e^ Kaplan–Meier test.

OS, Overall survival; CCI, Charlson Comorbidity Index; BMI, Body Mass Index; MLN, Metastatic lymph node; LVI, Lymphovascular invasion; PNI, Perineural invasion; LOS, Length of hospital stay.

The rate of surviving patients was 20.9% for those who received neoadjuvant treatment, and 39.6% for those who did not (*p* = 0.012). It was observed that patients who did not receive neoadjuvant therapy had a higher rate of 5-year OS than those who received neoadjuvant therapy (44.5% vs. 15.3%). The rate of advanced-stage patients was higher in the population who received neoadjuvant therapy (Table 2). No significant impact on survival was observed considering gender, Charlson Comorbidity Index (CCI), body mass index (BMI), type of surgical procedure or stage of differentiation.

**Table 2:** Disease staging of the patients considering neoadjuvant therapy status.

			pT stage		pN stage			pTNM stage	
			T1/T2	T3/T4	N1	N2	N3	IIA	III
Neoadjuvant therapy	No	*n*	12	84	34	27	35	36	60
		%	12.5%	87.5%	35.4%	28.1%	36.5%	37.5%	62.5%
	Yes	*n*	5	62	17	12	38	9	58
		%	7.5%	92.5%	25.4%	17.9%	56.7%	13.4%	86.6%

**Figure 2:**
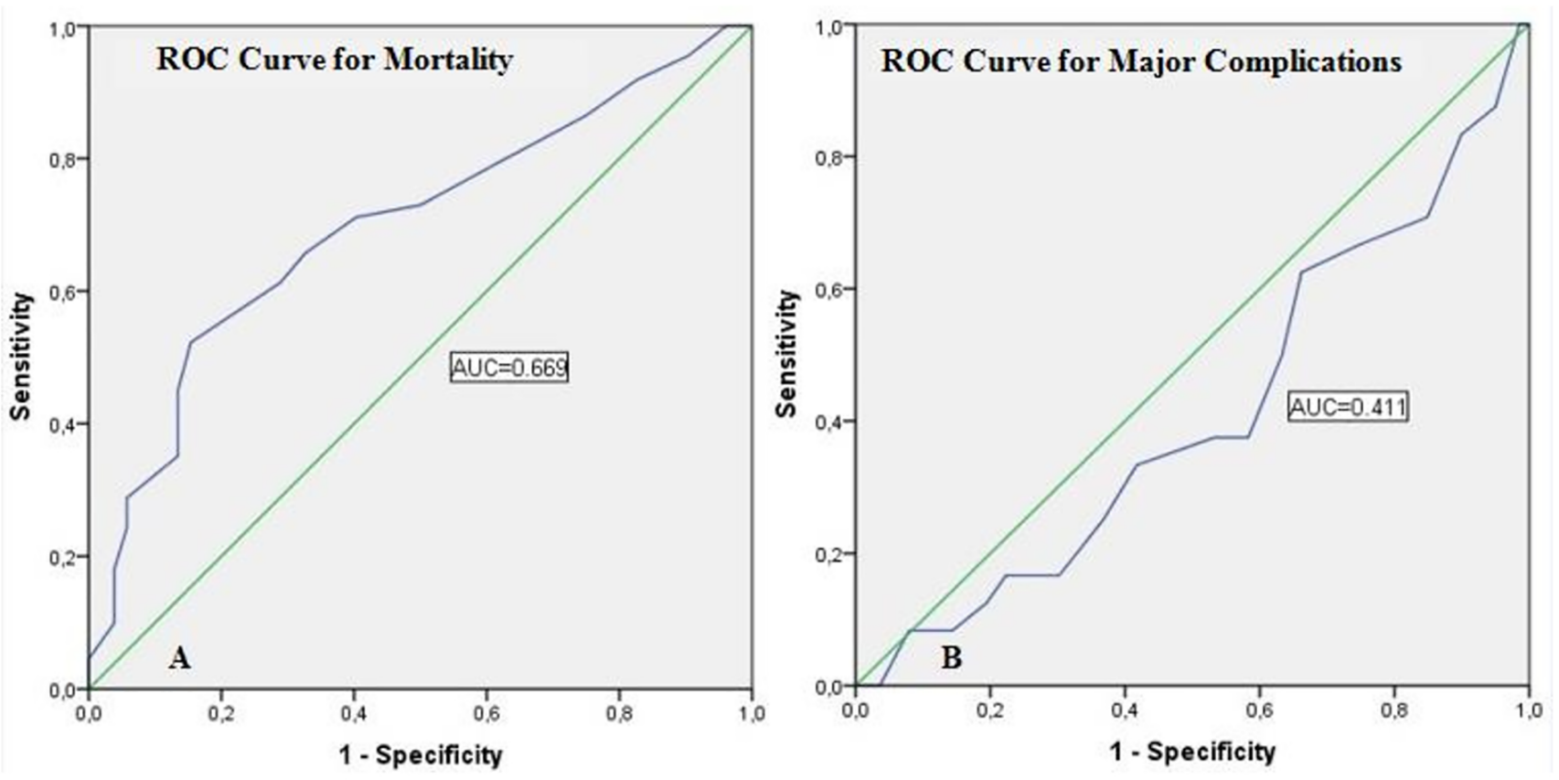
ROC analysis of MLN size in predicting mortality and major complications.

Assessment of the reliability of MLN size in predicting mortality and major complications with ROC curves was presented in Table 3. The cut-off value of MLN size predicting mortality was determined as 1.05 cm. The area under the curve (AUC) was 0.699, the sensitivity was 65.8%, and the specificity was 67.3% for this cut-off value (*p* < 0.001). On the other hand, the sensitivity and specificity of the MLN size cut-off value (1.05 cm), which predicts major complications, was quite low and was not statistically significant (*p* = 0.164) (Figure 2).

**Table 3:** Assessment of the metastatic lymph node size in predicting mortality and major complications with ROC curves .

	AUC	95% CI	Cutoff	Sensitivity	Specificity	Youden Index	*p-value*
MLN size (cm)^a^	0.699	0.615–0.782	1.05	65.8%	67.3%	0.331	**<0**.**001**
MLN size (cm)^b^	0.411	0.287–0.585	1.05	37.5%	41.7%	−0.208	0.164

^a^ for mortality status.

^b^ for major complications.

ROC, Receiver operating characteristic; AUC, Area Under Curve; CI, Confidence interval; MLN, Metastatic lymph node.

The relationship between cut-off value of metastatic lymph node size and clinicopathological features was presented in Table 4. Most of the patients (68.7%) who received neoadjuvant treatment had MLN size ≥1.05 cm (*p* = 0.004). Among the patients with MLN size ≥1.05 cm, 90.4% were in the pN3 group, and only 5.9% were in the pN1 group (*p* < 0.001). In addition, LVI positivity rate was 59.5% (*p* = 0.025) and PNI positivity rate was 62.5% (*p* < 0.001) in patients with MLN size ≥1.05 cm.

**Table 4:** Relationship between cut-off value of metastatic lymph node size and clinicopathological features.

Variables, *n* (%)		Metastatic lymph node size				*p-value*
		<1.05 cm (*n* = 73)		≥1.05 cm (*n* = 90)		
		*n*	%	*n*	%	
Age, years, median (IQR)	63 (56–71)	62 (51–66)	0.105^a^
Gender	Male	57	46.7%	65	53.3%	0.391^b^
	Female	16	39.0%	25	61.0%	
CCI	0–2	53	42.7%	71	57.3%	0.350^b^
	≥3	20	51.3%	19	48.7%	
BMI, kg/m^2^	<30	56	42.4%	76	57.6%	0.211^b^
	≥30	17	54.8%	14	45.2%	
Neoadjuvant	No	52	54.2%	44	45.8%	0.004^b^
	Yes	21	31.3%	46	68.7%	
Surgery	Total	41	48.8%	43	51.2%	0.287^b^
	Subtotal	32	40.5%	47	59.5%	
pT stage	T1/T2	10	58.8%	7	41.2%	0.219^b^
	T3/T4	63	43.2%	83	56.8%	
pN stage	N1	48	94.1%	3	5.9%	<0.001^b^
	N2	18	46.2%	21	53.8%	
	N3	7	9.6%	66	90.4%	
LVI	No	20	62.5%	12	37.5%	0.025^b^
	Yes	53	40.5%	78	59.5%	
PNI	No	25	71.4%	10	28.6%	<0.001^b^
	Yes	48	37.5%	80	62.5%	
Differention	Well	3	75.0%	1	25.0%	0.054^c^
	Moderately	28	57.1%	21	42.9%	
	Poorly	42	38.2%	68	61.8%	
Major Complications	No	58	41.7%	81	58.3%	0.059^b^
	Yes	15	62.5%	9	37.5%	
LOS, days, median (IQR)	8 (7–14)	9 (7–12)	0.717^a^
OS, estimated months	42.0% (87.9)	17.2% (71.8)	<0.001^d^

^a^ Mann–Whitney *U* test.

^b^ Pearson's Chi-Square test.

^c^ Likelihood ratio.

^d^ Kaplan–Meier analysis.

CCI, Charlson Comorbidity Index; BMI, Body Mass Index; LVI, Lymphovascular invasion; PNI, Perineural invasion; LOS, Length of hospital stay; OS, Overall survival.

**Figure 3:**
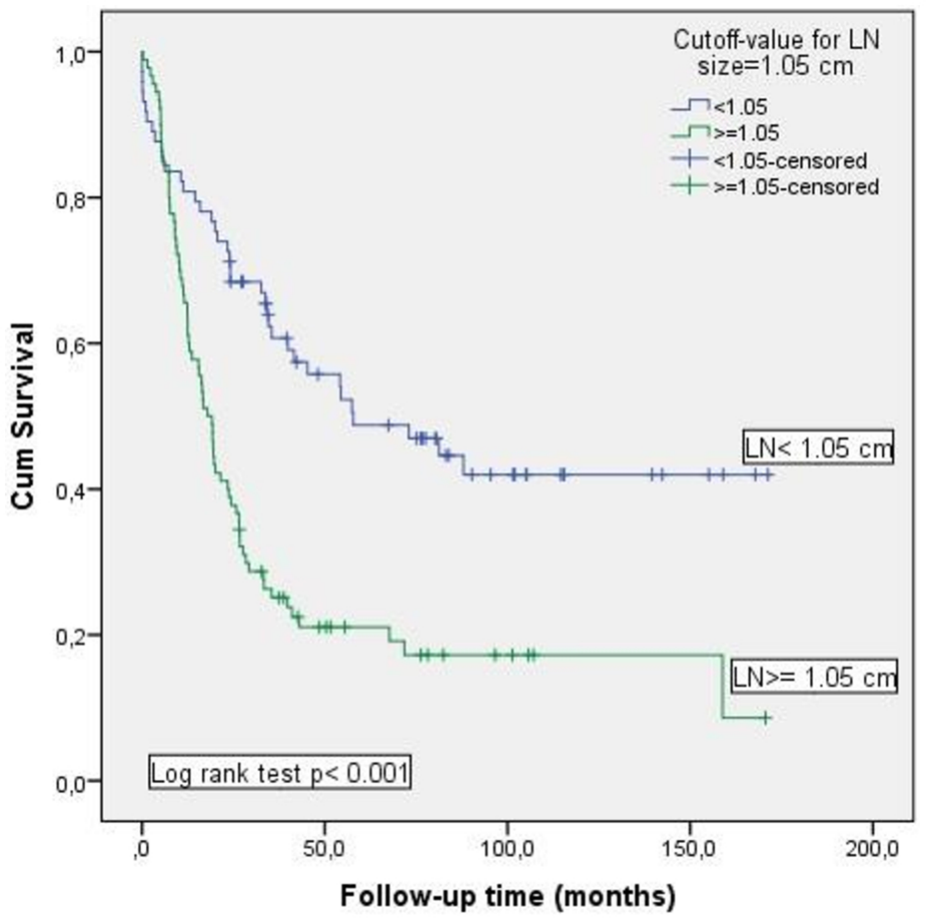
Kaplan–Meier analysis of the impact of metastatic lymph node size on overall survival.

The Kaplan–Meier method was used to analyze the role of MLN size on OS (Figure 3). Survival rate was significantly decreased in patients with MLN size ≥1.05 cm compared to those with MLN size <1.05 cm [17.2% vs. 42%; (*p* < 0.001)].

Results of multivariate logistic regression analysis of factors associated with mortality were given in Table 5. Age, receiving neoadjuvant therapy, pN stage, and MLN size ≥1.05 cm were found as independent risk factors for mortality. Among these, the most prominent risk factor was the diameter of MLN size (≥1.05 cm), and had nearly 3.5 times more negative impact on survival.

**Table 5:** Multivariate logistic regression analysis predicting mortality.

Variables	OR	95.0% CI	*p-value*
Age, years	1.029	1.012–1.046	0.001
Neoadjuvant, yes	2.167	1.450–3.240	<0.001
pT stage, T3/T4	1.758	0.794–3.889	0.164
pN stage, N2/N3	2.256	1.274–3.994	0.005
MLN size, ≥ 1.05 cm	3.584	2.030–6.328	<0.001
LVI, yes	1.311	0.751–2.290	0.340
PNI, yes	1.104	0.629–1.937	0.730

OR, Odds ratio; CI, confidence interval; LVI, Lymphovascular invasion; PNI, Perineural invasion; MLN, Metastatic lymph node.

## Discussion

Our study results showed that evaluation of the largest MLN size *via* the histopathological examination may provide valuable information predicting mortality in patients with stage II-III GC. MLN size may be considered a reliable prognostic factor in GC.

The importance of LN size in gastric cancer has been investigated decades ago. LNs with and without metastases in GC patients were examined and it was reported that LN size was not an important factor in the determination of metastasis (12). Later, studies conducted with MLNs showed that MLN size significantly affected prognosis of the patients with esophageal and colorectal cancers. Dhar et al. (7) reported the size of the largest MLN as the strongest independent predictor in a study of 187 patients with squamous cell carcinoma of the esophagus. Similarly, in a study in which a survival analysis of 311 colorectal cancer patients was performed, MLN size was found to be a strong prognostic variable in colorectal carcinoma (8). There was also a study in which the LN size was examined radiologically before surgery in GC. The size of the largest LN visualized on computed tomography (CT) was useful for predicting the MLN status of gastric cancer (13).

The first study in the literature investigating the largest MLN size in gastric cancer histopathologically and evaluating its effect on prognosis was conducted by Dhar et al. in 2003 (9). In that study, a total of 237 patients who had undergone surgery due to GC were included in the survival analysis. The largest MLN size was ranging from 0.3 to 3.0 cm and they determined a cut-off value of 7 mm for survival comparison. All tumors were classified using the 1997 The Union for International Cancer Control (UICC) pTNM categories; only patients with visceral metastases and distant lymph node metastases were excluded, all T and N stages were included. Results from this Japanese study demonstrated that MLN size was an independent risk factor in determination of OS and disease-free survival (DFS). Furthermore, it was also revealed that MLN size may supplement the UICC nodal classification system by stratifying node positive patients (9). Another similar study which was conducted in Korea evaluated the effect of the largest MLN size on prognosis in GC (10). Using a categoric cut-off value of 2 cm, they found that OS and DFS were significantly better in patients with smaller (<2 cm) MLN size. A large MLN (≥2 cm) had been reported to be an independent predictor of poor prognosis in patients with node-positive gastric carcinoma (10). A cut-off value of MLN size for survival comparison was 1.05 cm and largest MLN size was ranging from 0.3 to 2.3 cm in our study. Even though with different cut-off values, we found similar results to previously reported studies that the largest MLN size may be an important prognostic factor for OS in GC with lymph node metastasis.

Nodal involvement (*N* stage) was one of the prognostic factors for patients eligible for surgery in GC and used in the most commonly applied staging systems (2). It was reported as an independent prognostic factor since there's a close relationship between lymph nodes, tumor stage, and prognosis (14). In our MLN size <1.05 cm group, 48 patients were in the pN1 stage and 7 patients were in the pN3 stage. The MLN size ≥1.05 cm group included 3 patients at pN1 stage and 66 patients at pN3 stage. In the patient population included in our study, as pN stage increased, we observed a significant increase in MLN size and a significant decrease in survival. This result is in line with the published literature.

It was reported that patients with major complications required longer hospital stays and these complications had a negative effect on survival outcomes (15). As expected, the length of hospital stay was longer in patients with major complications in the present study. In addition, patients with larger MLN size had longer hospital stays in our study. We evaluated the post-operative complications that were not investigated in the previous studies. However, we did not detect a relationship between the largest MLN size and the presence of major complications.

The absence or presence of LVI and PNI was important indicators of invasive tumors and they provide valuable information regarding survival outcomes in GC. They were associated with a higher number of positive LNs, pathologically more advanced tumors, and shorter OS and DFS (16). In our study, LVI and PNI positivity were prominent in patients with MLN size ≥1.05 cm and the positivity rate of LVI and PNI was significantly higher in the deceased patient group. Therefore, we consider that our results are in line with the literature.

Limitations of the study are its retrospective design and relatively small sample size. Another limitation relates to our analysis is disease free survival. Since recurrence data was not set as one of the endpoints, these data had not been assessed systematically and were incomplete. However, there were a limited number of previous studies in this area. In contrast to Dhars' and Cheongs' studies, not using categorical cut-off and preventing stage bias by including a limited pathological stage group are the strengths of our study. This paper expresses a different perspective on the relationship between postoperative complications and the largest MLN size.

## Conclusion

Our study results indicated that the largest MLN size was an independent risk factor for survival and a cut-off value of 1.05 cm in MLN size had prognostic value in surgically treated stage II-III GC patients. However, we did not find a relationship between the largest MLN size and the presence of major complications. In the light of these results, a review of N-stage subgroups of TNM staging may be considered. Further multicenter studies with large sample size are required to confirm our study results.

## Data Availability

The original contributions presented in the study are included in the article/Supplementary Material, further inquiries can be directed to the corresponding author/s.
